# Bovine Leukemia Virus Small Noncoding RNAs Are Functional Elements That Regulate Replication and Contribute to Oncogenesis In Vivo

**DOI:** 10.1371/journal.ppat.1005588

**Published:** 2016-04-28

**Authors:** Nicolas A. Gillet, Malik Hamaidia, Alix de Brogniez, Gerónimo Gutiérrez, Nathalie Renotte, Michal Reichert, Karina Trono, Luc Willems

**Affiliations:** 1 Molecular and Cellular Epigenetics, Interdisciplinary Cluster for Applied Genoproteomics (GIGA), University of Liège, Liège, Belgium; 2 Molecular and Cellular Biology, Gembloux Agro-Bio Tech, University of Liège, Gembloux, Belgium; 3 Instituto de Virología, Centro de Investigaciones en Ciencias Veterinarias y Agronómicas, Castelar, Argentina; 4 National Veterinary Research Institute, Pulawy, Poland; University of Texas at Austin, UNITED STATES

## Abstract

Retroviruses are not expected to encode miRNAs because of the potential problem of self-cleavage of their genomic RNAs. This assumption has recently been challenged by experiments showing that bovine leukemia virus (BLV) encodes miRNAs from intragenomic Pol III promoters. The BLV miRNAs are abundantly expressed in B-cell tumors in the absence of significant levels of genomic and subgenomic viral RNAs. Using deep RNA sequencing and functional reporter assays, we show that miRNAs mediate the expression of genes involved in cell signaling, cancer and immunity. We further demonstrate that BLV miRNAs are essential to induce B-cell tumors in an experimental model and to promote efficient viral replication in the natural host.

## Author Summary

The recent discovery of miRNA expression by retroviruses is a matter of active debate and some controversy. Several retroviruses including human immunodeficiency virus (HIV) potentially encode functional small non-coding RNAs that are nevertheless undetectable in vivo. While these miRNAs regulate multiple host and viral processes in vitro, their contribution to infection and pathogenesis remains largely unknown. Using next-generation sequencing and reverse genetics, we provide mechanistic and functional evidence demonstrating that BLV miRNAs are functional genetic elements that regulate replication and contribute to oncogenesis in suitable animal models.

## Introduction

Virally encoded miRNAs were first identified in DNA viruses. Most of these miRNAs are generated from endonucleolytic cleavage of long viral transcripts. Retroviruses were initially assumed not to encode miRNAs because of the potential self-cleavage of their RNA genome. Human immunodeficiency virus (HIV), West Nile virus (WNV), Dengue virus (DENV), hepatitis A virus (HAV) and Ebola virus (EBOV) potentially express small non-coding RNAs [1–4]; however, no significant amounts of viral miRNAs have been identified by high-throughput sequencing of RNA from cultured cells infected with these different viruses [5–7]. Recently, four retroviruses, i.e., bovine leukemia virus (BLV), bovine foamy virus (BFV), avian leucosis virus (ALV-J) and simian foamy virus (SFV) were shown to express viral miRNAs in infected cells [8–11]. ALV-J encodes a single viral miRNA (XSR) from a Pol II-transcribed precursor that is processed by the canonical biogenesis pathway. ALV-J thus tolerates some Drosha and Dicer cis-cleavage of the viral genome. In contrast, BLV and BFV massively express viral miRNAs driven by Pol III promoters embedded within their proviral genome. These viruses avoid unproductive cleavage of their genomic RNA because only the subgenomic Pol III transcripts are processed into miRNAs. The BFV long terminal repeat U3 region is transcribed into a 122-nucleotide pri-miRNA that is subsequently cleaved into two pre-miRNAs and processed in three viral miRNAs. The BLV genome encodes a cluster of five miRNA hairpins from a proviral region lacking significant open reading frames located just 3' to the envelope gene. These hairpins undergo maturation into ten miRNAs in a Drosha-independent pathway [8,12,13].

BLV naturally infects the bovine species (*Bos taurus*), zebu (*Bos indicus*) and water buffalo (*Bubalus bubalis*). BLV infection remains mostly asymptomatic in a large majority of infected animals. Only approximately one-third of BLV-infected cows will develop benign lymphocytosis. In a minority of cases (approximately 5%), BLV infection leads to B-cell leukemia/lymphoma after long latency periods (7–10 years). Experimental inoculation of sheep with BLV recapitulates the different steps of oncogenesis with higher frequencies (up to 100%) and relatively short latency periods (approximately 2 years) [14]. Remarkably, BLV persists and replicates in vivo in the absence of significant levels of viral mRNA transcription [15–17]. In contrast, BLV miRNAs are massively expressed in the infected cells, representing up to approximately 40% of all cellular miRNAs [12]. Because ribonucleic acids are likely less immunogenic than viral antigens, BLV miRNAs could thus modify the cell fate and concomitantly escape from immune recognition.

Although miRNAs encoded by BLV are massively expressed, their biological significance in viral replication and pathogenesis is still unknown. In this report, we specifically addressed this question in the BLV system using a reverse genetics approach.

## Results

### BLV miRNAs modulate the expression of genes involved in oncogenesis, cell signaling, apoptosis and immune response

**Fig 1:**
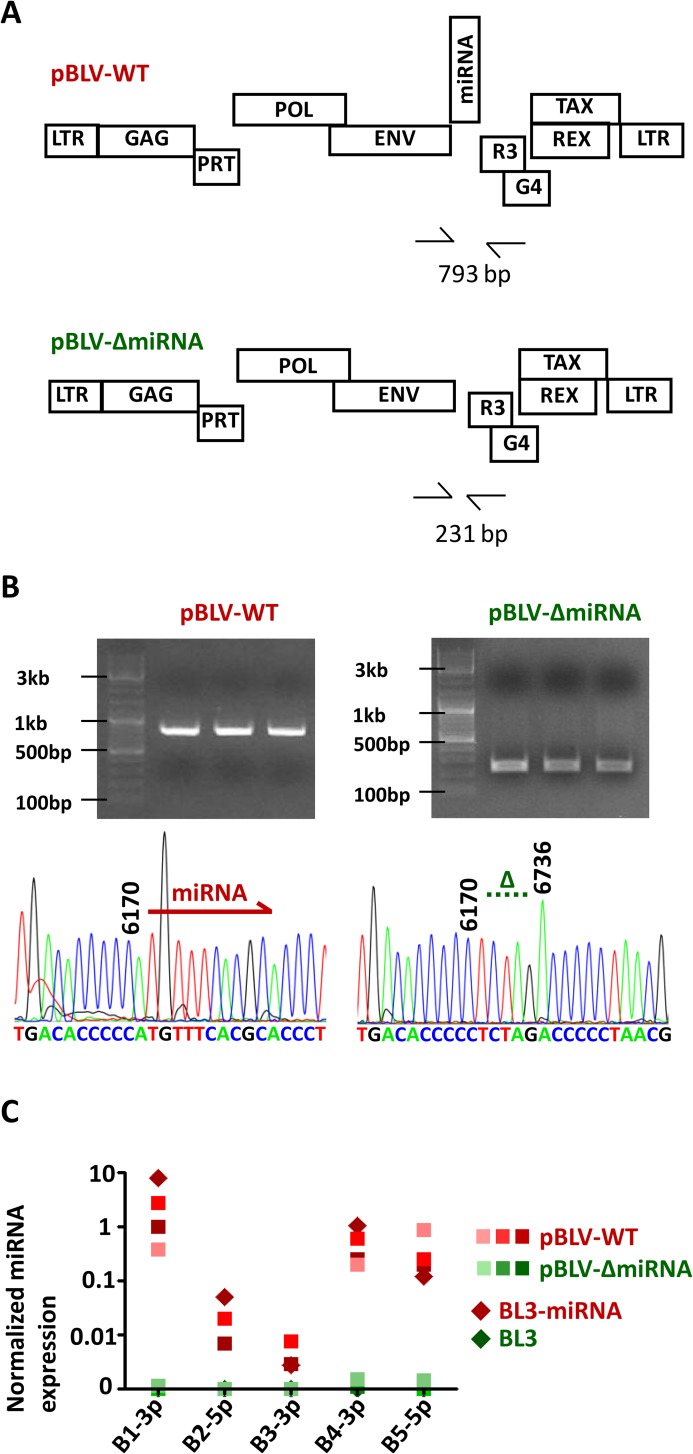
**miRNA-deleted BLV virus and BLV miRNAs-expressing bovine B-cell line (A) A modified version (pBLVΔ-miRNA) of the wild type BLV provirus (pBLV-WT) was generated by deleting the region encoding the miRNAs.** **(B)** Upon inoculation of the calves, the integrity of the miRNA sequences was verified by nested PCR (upper panels) and Sanger sequencing (lower panels). 793 bp and 231 bp correspond to the amplicon lengths of pBLV-WT and pBLVΔ-miRNA, respectively. **(C)** miRNA expression levels were measured by quantitative RT-qPCR in miRNA-transduced BL3 cells (BL3-miRNA) and in primary PBMCs from WT and Δ-miRNA-infected calves.

To investigate the role of the miRNAs in the natural host, a BLV provirus isogenic to a wild-type molecular clone but devoid of the intergenic sequences located between the env and R3 genes (pBLV-ΔmiRNA) was constructed (Fig 1A) and inoculated into 3 calves. All three animals produced specific antibodies against BLV gp51 envelope protein and became persistently infected by the deleted virus, demonstrating that the miRNAs are dispensable for infectivity. Serial PCR amplifications of peripheral blood mononuclear cell (PBMC) DNA with primers flanking the miRNA region and subsequent sequencing confirmed that pBLV-ΔmiRNA was indeed infectious in vivo (Fig 1B). Quantitative RT-qPCR further revealed the lack of viral miRNA expression in these animals (B1-3p, B2-5p, B3-3p, B4-3p and B5-5p), as in a control bovine B-lymphocyte cell line (BL3) (Fig 1C). In contrast, the miRNA copy numbers measured in primary PBMCs from wild-type infected calves (Bo-WT) were similar to the levels of BL3 cells transduced with a lentiviral vector expressing the BLV miRNAs (BL3-miRNA, Fig 1C).

**Fig 2:**
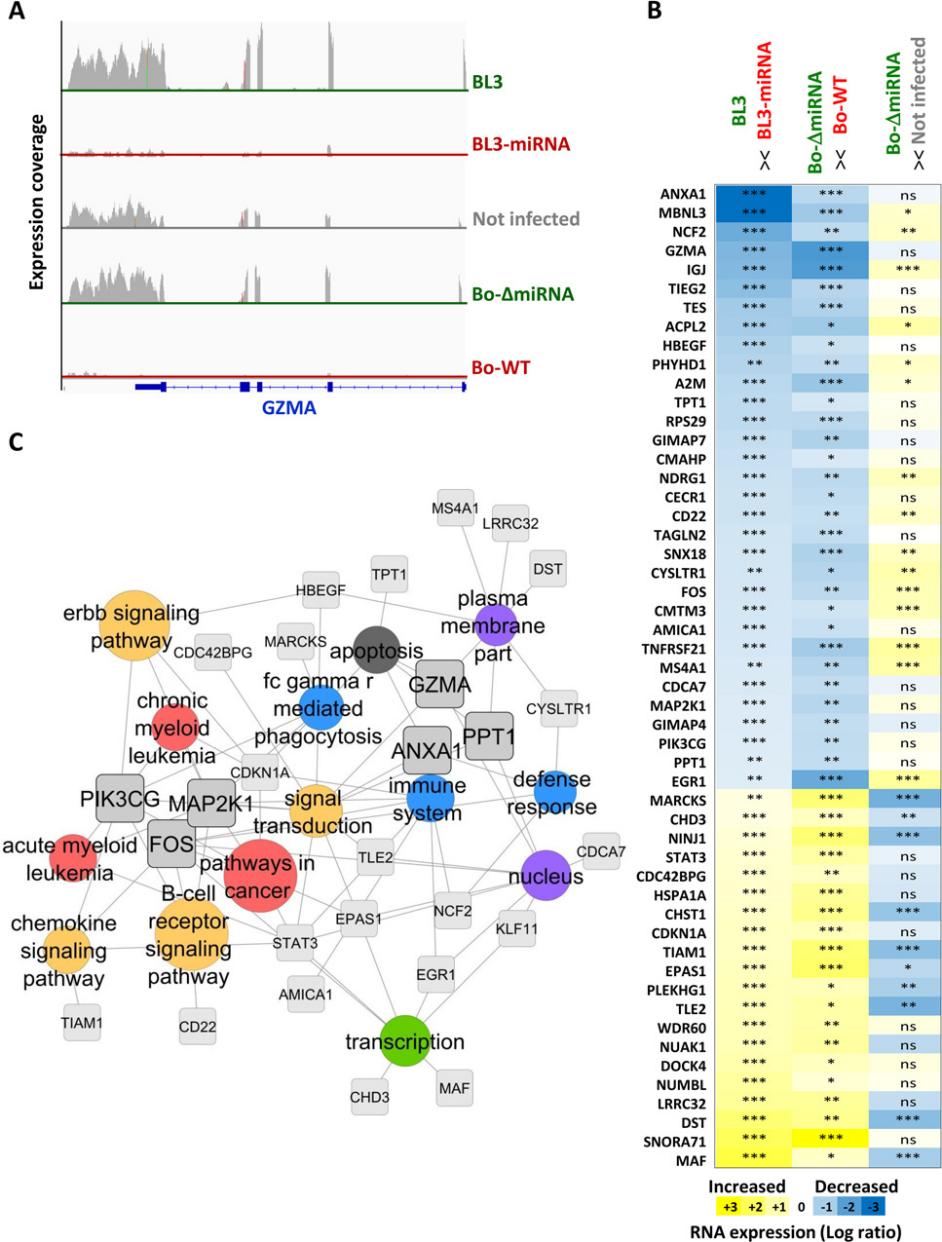
Transcriptome changes induced by BLV miRNAs. **(A)** The transcriptomes of BL3 and BL3-miRNA cells and primary PBMCs from mock-, pBLVΔ-miRNA or pBLV-WT-infected calves (Not infected, Bo-Δ-miRNA and Bo-WT, respectively) were determined by RNA-Seq. GZMA transcriptional activity is illustrated using the Integrative Genomics Viewer (IGV) software from the Broad Institute. **(B)** Changes in gene expression induced by viral miRNAs. The first column (BL3 > < BL3-miRNA) lists the transcripts that are significantly deregulated in BL3 cells compared to BL3-miRNA. The second column (Bo-ΔmiRNA > < Bo-WT) compares the transcripts that are significantly modulated in primary PBMCs of the calves infected with pBLV-WT and pBLVΔ-miRNA. The third column (cow-ΔmiRNA > < not infected) reports the differences in the expression levels between Bo-ΔmiRNA PBMCs and uninfected controls. A colored scale with increasing shades of blue indicating depletion and increasing shades of yellow indicating enrichment is shown on the bottom of panel E. P-values were calculated using Cuffdiff software (NS for not significant,* p< 0.05, ** p< 0.01, *** p< 0.001). Sequencing data results from biological triplicates in each group. **(C)** Pathway enrichment analysis of miRNA-induced transcriptional modifications. The genes significantly deregulated by the viral miRNAs were clustered by ontology using MSigDB. The gene regulation network was constructed using Cytoscape software. The circle size is proportional to enrichment of the annotation. FDR q-value threshold for gene ontology annotation was set to 0.05.

Because a minimal heptameric seed sequence is sufficient for activity, the ten BLV miRNAs are predicted to substantially modify the host transcriptome. To address species-specificity, cell type adequacy and biological relevance, gene expression was characterized in parallel in primary PBMCs and miRNA-transduced BL3 lymphocytes. High-throughput RNA sequencing was performed with pLenti-miRNA-transduced BL3 cells (BL3-miRNA) or control pLenti-transduced BL3 (BL3) and with PBMCs from wild-type BLV (Bo-WT), pBLV-ΔmiRNA (Bo-ΔmiRNA) or mock-infected calves. Fig 2A illustrates the expression coverage profile corresponding to the granzyme A (GZMA) locus. GZMA mRNA was expressed in control lymphocytes (BL3) but not in pLenti-miRNA-transduced BL3 cells (BL3-miRNA). Consistently, GZMA transcripts were present in PBMCs from pBLV-ΔmiRNA-inoculated animals (Bo-ΔmiRNA) and uninfected controls but were almost undetectable in Bo-WT calves (Fig 2A). The combined analysis of BL3 cells and PBMCs thus identified mRNAs associated with the presence of the miRNAs in B lymphocytes and in vivo. Panel B of Fig 2 provides the top list of mRNAs that match these criteria. Gene ontology analysis using MSigDB software (Molecular Signature DataBase from the Broad Institute) identified a network of the most enriched pathways that included cell signaling, cancer genes and immune response (Fig 2C).

### FOS, GZMA and PPT1 are biologically relevant miRNA targets

**Fig 3:**
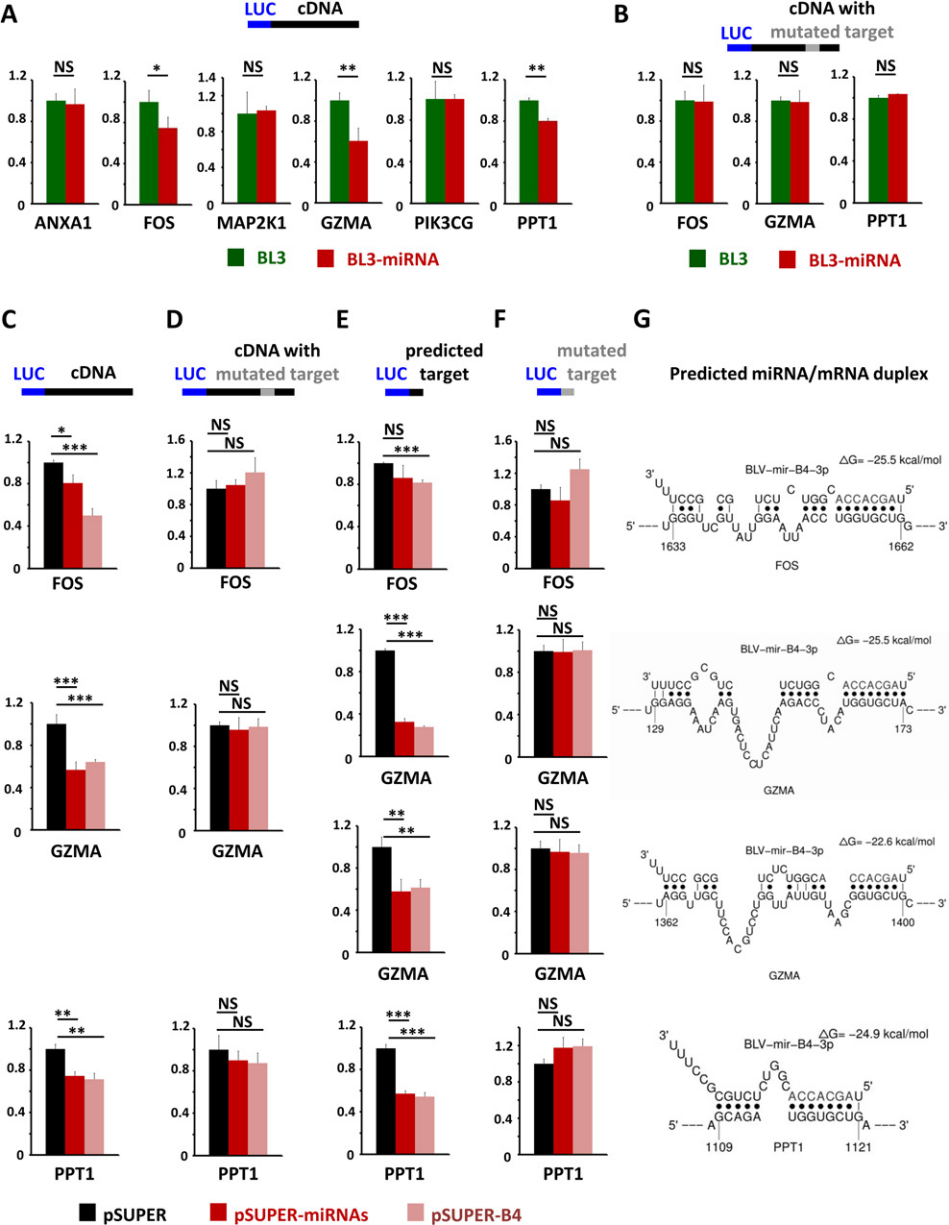
Validation of RNA targets by luciferase reporter assays. A series of psiCHECK2 luciferase reporter plasmids were constructed by insertion of the full length cDNAs of ANXA1, FOS, MAP2K1, GZMA, PIK3CG and PPT1 **(A,C)**, the full length cDNAs mutated in the miRNA predicted target sequence(s) **(B,D)**, the miRNA predicted target sequence **(E)** and a mutated predicted target sequence **(F)**. Reporter plasmids were transfected in BL3 or BL3-miRNA cells and luciferase activity was determined at 24 hours (A,B). Y axis are relative luciferase units. Reporter plasmids were also co-transfected with expression vectors for all 10 viral miRNAs (pSUPER-miRNAs) or for miRNA-B4 only (pSUPER-B4) in HEK 293T cells and luciferase activity was determined at 24 hours (**C-F**). **(G)** Prediction of miR-B4-3p duplexes with cFOS, GZMA and PPT1 transcripts using the STarMir program. NS (not significant), * p< 0.05, ** p< 0.01, *** p< 0.001 according to Student's t-test. Error bars are standard deviations.

The expression networks of statistically significant and biologically relevant genes revealed intramodular hubs in consensus modules (Fig 2C). Based on their connection frequency, the following six hub genes were selected for further functional analysis (boxes on Fig 2C): PPT1 (palmitoyl-protein thioesterase 1), ANXA1 (annexin A1), GZMA (granzyme A), PIK3CG (phosphatidylinositol-4,5-bisphosphate 3-kinase, catalytic subunit gamma), FOS (FBJ murine osteosarcoma viral oncogene homolog) and MAP2K1 (mitogen-activated protein kinase kinase 1 or MEK1). BLV miRNA functionality was evaluated in BL3 cells by reporter constructs containing gene cDNAs cloned downstream of the Renilla luciferase gene. BLV miRNAs silenced the luciferase activity of reporter plasmids containing the full-length cDNAs of FOS, GZMA and PPT1 (compare BL3-miRNA with BL3 on Fig 3A). As a control for specificity, luciferase expression of a backbone reporter lacking cDNAs was unaffected by BLV miRNAs (S3 Fig). In contrast, ANXA1, MAP2K1 and PIK3CG reporters were not down-regulated under similar conditions, indicating that these genes were not direct miRNA targets (Fig 3A). The FOS, GZMA and PPT1 mRNAs were predicted by Sfold and STarMiRNA software to be direct interactors of miRNA B4-3p (Fig 3G and S1 Fig). Mutation of the predicted miRNA binding sites in the full-length cDNAs of FOS, GZMA and PPT1 abrogated miRNA silencing (Fig 3B). To test the selective effect of BLV miRNA B4 on the FOS, GZMA and PPT1 mRNAs, HEK293T cells were transfected with a pSUPER plasmid encoding only miRNA B4 (Fig 3C–3F). The levels of miRNA expression achieved in HEK293T cells were biologically relevant as they were similar to those measured in PBMCs (S2 Fig). BLV miRNA B4 silenced the luciferase activity of reporter plasmids containing the full-length cDNAs of FOS, GZMA and PPT1 (compare pSUPER with pSUPER-B4 on Fig 3C). Mutation of the predicted miRNA binding sites in the full-length cDNAs of FOS, GZMA and PPT1 abrogated the miRNA B4 silencing (Fig 3D). Insertion of the predicted target (Fig 3E) but not of a mutated version (Fig 3F) in the 3'UTR of the luciferase gene recapitulated the silencing activity of the miRNAs.

These functional reporter assays thus identified direct (FOS, GZMA and PPT1) and indirect (ANXA1, MAP2K1 and PIK3CG) targets among biologically relevant genes whose expression is affected by BLV miRNAs in vivo.

### BLV miRNAs are dispensable for infection but contribute to viral replication in the natural host

**Fig 4:**
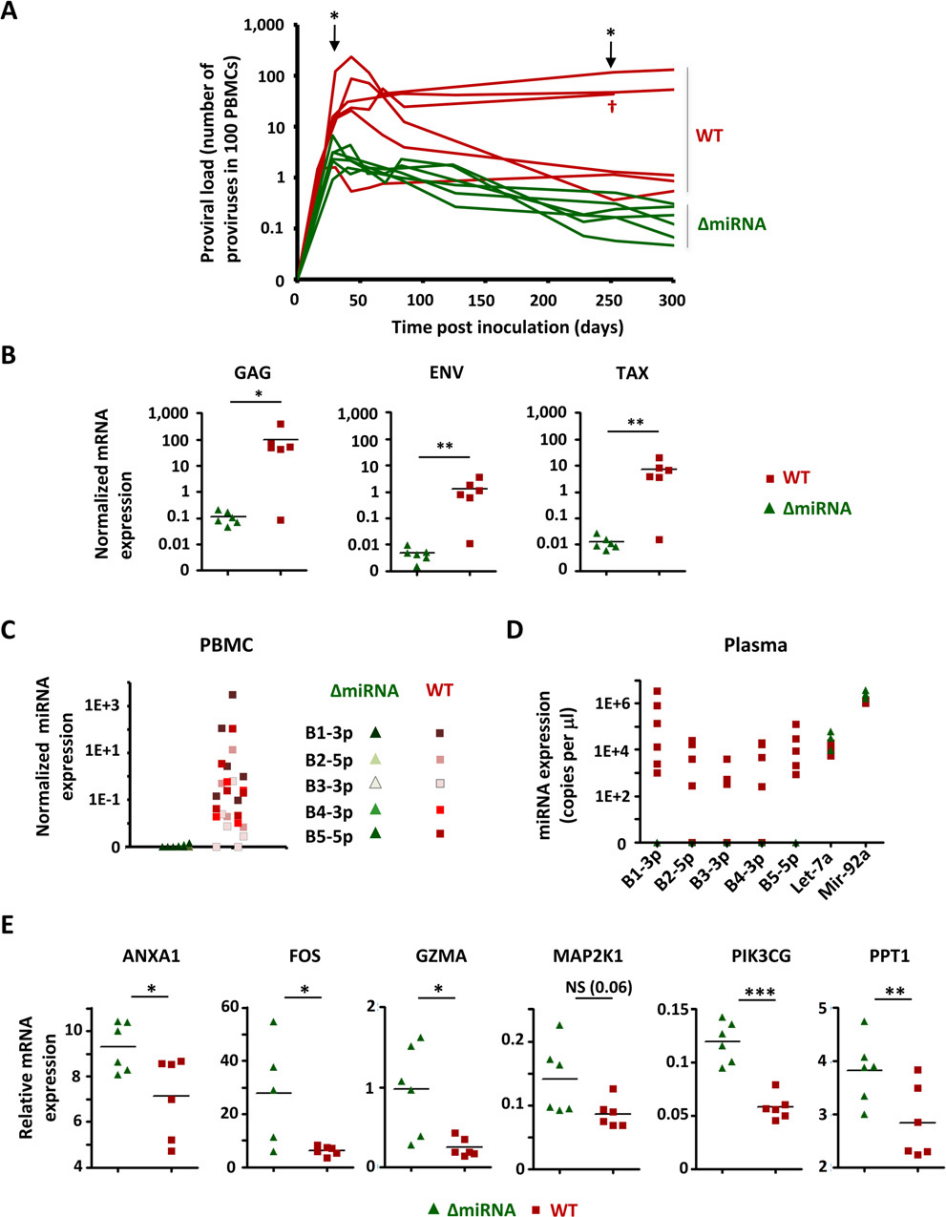
Viral transcription and replication in the bovine natural host. **(A)** Calves were inoculated at day 0 with the pBLVΔ-miRNA wild-type provirus (WT) or with the isogenic strain deleted of the miRNAs (ΔmiRNA). Proviral loads (number of copies in 100 PBMCs) were determined by quantitative real time PCR. * (p< 0.05) means statistically significant according to Mann Whitney test. One BLV-infected calf died (†). **(B)** PBMCs from pBLVΔ-miRNA and pBLV-WT-infected calves were cultivated *ex vivo* for 24 h. The levels of GAG, ENV and TAX mRNAs were determined by RT-qPCR and normalized to HPRT. * (p< 0.05) or ** (p< 0.01) means significant or highly significant, respectively, according to the Mann Whitney test. **(C)** BLV miRNAs (B1-3p, B2-5p, B3-3p, B4-3p and B5-5p) in primary PBMCs were quantified by RT-qPCR and normalized to HPRT. **(D)** Plasmatic levels (copy numbers in 1 μl of plasma) of viral and host (Let-7a and Mir-92a) miRNAs were titrated by RT-qPCR. **(E)** RNA was extracted from pBLV-WT (red) or pBLVΔ-miRNA (green)-infected PBMCs and analyzed by RT-qPCR for ANXA1, FOS, GZMA, MAP2K1, PIK3CG and PPT1 expression. Statistical significance was determined by the Mann Whitney test: NS (not significant), * p< 0.05, ** p< 0.01, *** p< 0.001.

To evaluate the role of the miRNAs in viral fitness, 12 calves were inoculated either with the wild-type molecular clone or with the recombinant pBLV-ΔmiRNA. The proviral loads profile indicated that the viral burden at 28–30 days post-inoculation was significantly lower in pBLV-ΔmiRNA infected animals compared to wild-type levels (Fig 4A Mann-Whitney test, p = 0.04). The integrity of the miRNAs was also important to maintain stable proviral loads in the long term (i.e., at 252–254 days on Fig 4A; Mann Whitney test, p = 0.03). Nested PCR and sequencing indicated the absence of reversion of the mutation at any time post-infection (S4 Fig). Consistently, spontaneous ex-vivo transcription of viral mRNAs was reduced in the animals infected with pBLV-ΔmiRNA compared to wild-type levels (Fig 4B Mann Whitney test, p = 0.009, p = 0.004 and p = 0.002 for GAG, ENV and TAX, respectively). miRNAs were abundantly expressed in PBMCs (Fig 4C) as well as in the plasma (Fig 4D) of wild-type BLV-infected calves and correlated with proviral loads but were undetectable in the pBLV-ΔmiRNA deletants. The expression of miRNA-regulated transcripts ANXA1, FOS, GZMA, MAP2K1, PPT1 and PIK3CG further validated the transcriptomic profiling data in a larger series of animals (Fig 4E).

Together, these observations demonstrate that ablation of the BLV miRNAs impacts replication fitness in the natural host.

### Suppression of oncogenicity in the absence of BLV miRNAs

**Fig 5:**
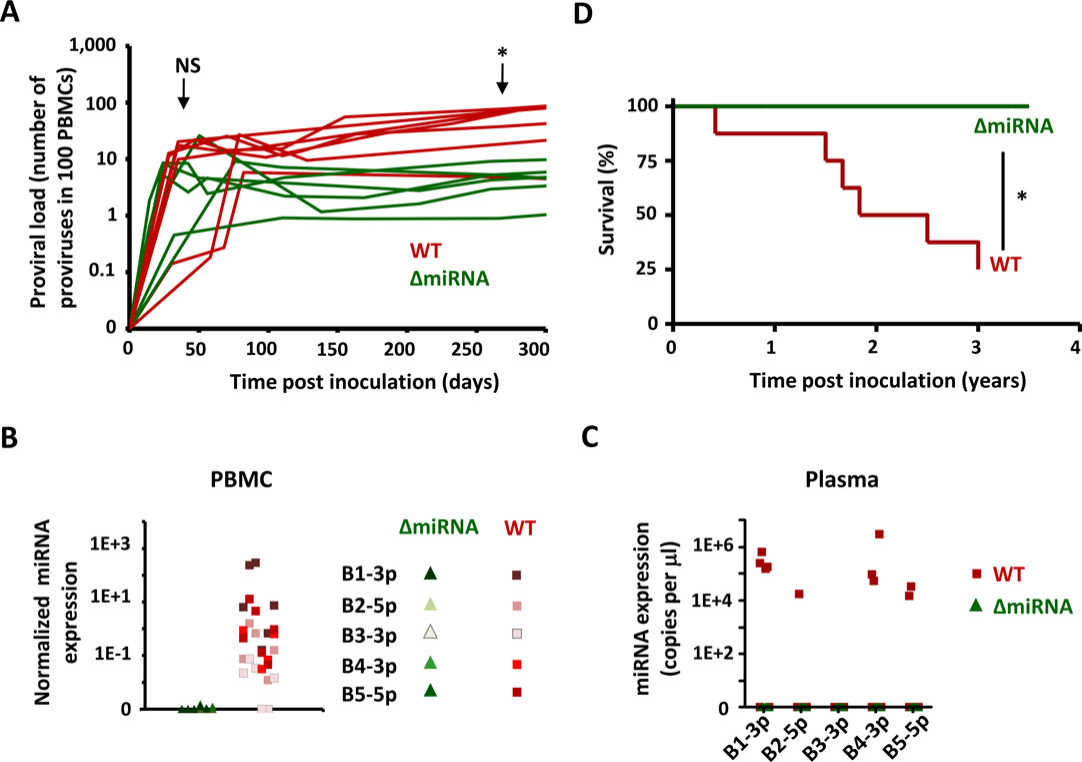
Viral replication and pathogenesis in experimentally inoculated sheep. **(A)** Sheep were inoculated at day zero with pBLV-WT (red) or pBLVΔ-miRNA (green). Proviral loads (number of copies in 100 PBMCs) were determined by real time PCR. NS (not significant) and * (p< 0.05) according to the Mann Whitney test. **(B)** BLV miRNAs (B1-3p, B2-5p, B3-3p, B4-3p and B5-5p) in primary PBMCs were titrated by real time quantitative PCR and normalized to HPRT. **(C)** BLV miRNAs (copies in 1 μl of plasma) were titrated and determined by RT-qPCR. **(D)** Survival curves of sheep infected with pBLVΔ-miRNA (n = 6 in green) or wild-type BLV virus (n = 7 in red). * (p< 0.05) according to the Logrank test.

Due to the low frequency of tumor development and the long latency period, the evaluation of the role of miRNAs in oncogenesis in the bovine species would take decades. Although sheep are not naturally infected by BLV, this model recapitulates the main characteristics of bovine leukemia-lymphoma and allows one to address this important question. Replication efficacy of the wild-type and pBLV-ΔmiRNA viruses was initially similar during early stages post-inoculation (NS, Fig 5A). At later times, the miRNA deletant was unable to maintain wild-type replication (*p = 0.04 according to the Mann Whitney test). The levels of miRNA expression in PBMCs and in the plasma were similar to those measured in the calves (Fig 5B and 5C). Since BLV miRNAs are abundantly expressed in the plasma, GZMA transcription could be affected in other cell types (CTLs and NK cells). Cell sorting and RT-qPCR demonstrated that miRNA-associated GZMA down-regulation is restricted to the B cell lineage (S4 Fig). As in the bovine species, replication of the pBLV-ΔmiRNA deletant in sheep occurred in the absence of reversion (S5 Fig). Fifty percent of the sheep infected with wild-type BLV developed leukemia-lymphoma within 22 months, confirming previous rates reported in the literature (Fig 5D) [18]. Compared to the high incidence of leukemia-lymphoma in the sheep inoculated with parental BLV, none of the animals infected with pBLV-ΔmiRNA displayed any clinical sign of oncogenicity (Fig 5D).

These data thus reveal a dramatic suppression of oncogenicity subsequent to the loss of BLV miRNAs.

## Discussion

The high abundance of BLV miRNAs in tumors suggested a possible role in viral replication and oncogenesis [8,12]. In this report, we have provided mechanistic and functional evidence demonstrating that the BLV miRNAs modify host gene expression to promote viral persistence and induce pathogenicity. The goal of the transcriptomic analysis was to identify the global changes associated with the BLV miRNAs in the natural host. The rationale was based on the merged analysis of a well-characterized cell system (BL3 bovine cells transduced with miRNAs) and primary PBMCs isolated from infected cows. The main reason of this strategy is that unbiased isolation of BLV-infected B cells from an infected animal is technically still impossible. Therefore, RNA sequencing was performed in parallel with miRNA or mock-transduced BL3 cells and primary PBMCs from cows inoculated with wild-type or recombinant pBLV-ΔmiRNA viruses. The objective was not to produce an exhaustive list of direct RNA targets but rather monitor the global effect of the miRNAs. High-throughput sequencing of RNA isolated from immunoprecipitated Ago-miRNA-mRNA complexes would identify only direct BLV miRNA targets. Transcriptomic profiling by high throughput RNA sequencing identified genes whose expression is affected by miRNAs in B-lymphocytes and primary PBMCs (Fig 2B). This approach revealed a list of biologically relevant genes that are directly (GZMA, FOS, PPT1) or indirectly (ANXA1, MAP2K1 and PIK3CG) targeted by the BLV miRNAs (Fig 3). In contrast, peroxidasin homolog (PXDN) identified by miRNA/RNA duplex prediction analyses and reporter assays was not a biologically relevant miRNA target in vivo [8,12]. Similarly, tumor suppressor HMGbox transcription factor 1 (HBP1) whose expression is halved in sheep tumor cells [12] is unaffected in bovine B-lymphocytes expressing the BLV miRNAs (S6 Fig). Gene ontology analysis revealed intramodular hubs in two consensus modules that relate to apoptosis and immunity (GZMA, PPT1 and ANXA1) or cell signaling and oncogenesis (FOS, MAP2K1 and PIK3CG). In particular, the serine protease GZMA expressed mostly by natural killer (NK) cells and cytotoxic T-lymphocytes (CTL) but also by B cells under inflammatory conditions [19], induces caspase-independent apoptosis [20,21]. Knockout of GZMA in mouse models sensitizes to the animals viral infections, indicating a central role in immunity [22]. Inactivation of GZMA by Kaposi's sarcoma-associated herpes virus (KSHV) miRNA-K12-6 further supports the significance of this target in other viral life cycles [23]. Our data (S4 Fig) show that GZMA expression is reduced by BLV miRNAs in B-lymphocytes but not in non-B cells. It remains nevertheless possible that microRNAs are transiently transferred via the immunological synapse upon contact with CTL or NK cells. Another important BLV miRNA target is FOS, which mediates the primary response to B-cell receptor signaling upon dimerization with c-JUN in the AP1 complex [24–26]. Interestingly, Tcl1-directed inhibition of AP-1 transcriptional activity is associated with a CLL-like disease in mice [27]. Similarly, transgenic mice overexpressing miR-29, a cellular miRNA sharing the BLV miRNA B4-3p seed sequence and repressing the FOS transcript [28], develop B-cell tumors [29]. FOS expression is also repressed by KSHV-encoded miRNA-K12-11 in lymphoma B-cells [23,30], illustrating common pathways of virus-induced oncogenesis. A third hub transcript that directly interacts with BLV miRNAs is PPT1, a glycoprotein involved in the catabolism of lipid-modified proteins during lysosomal degradation. PPT1 removes thioester-linked fatty acyl groups from cysteine residues and modulates tumor necrosis factor alpha (TNFα) signaling [31]. Because PPT1-deficient fibroblasts are partially resistant to TNF-induced cell death, BLV miRNAs could promote survival via this pathway [31]. RNA sequencing also revealed a series of genes whose expression is modified by pathways not involving direct seed-to-target interactions such as ANXA1, a mediator of macrophage phagocytosis. Together, the experimental data from this report and evidence from the literature highlight common mechanisms of transformation shared by other DNA viruses or those involved in human leukemia.

Assessing the role of viral miRNAs in cell lines faces objections of biological relevance in vivo. Transduction of BLV miRNAs into the BL3 cell line was not associated with alterations of cell proliferation or apoptosis (S7 Fig). BLV miRNAs do not directly impact on viral mRNAs or proteins in vitro (S8 Fig). In contrast, ablation of the miRNAs had significant effects on viral replication in the natural host (Fig 4). Compared to the wild-type controls, the proviral loads associated with miRNA deletants were indeed significantly lower during the primary infection and decreased regularly in the long-term. Whether miRNAs deletion will ultimately lead to an abortive infection remains to be determined but is predicted to require extensive periods of several years. Notwithstanding, a decrease in viral burden correlates with a reduction in viral transmission as well as pathogenicity. Consistently, our preliminary evidence indicates that the miRNA deletant does not transmit from cow-to-calf (S9 Fig). Considering the low frequency (5%) and long latency period (7–10 years) of tumor development in the natural host, assessing the role of the miRNAs in the bovine species is an interesting but almost unanswerable question. Nevertheless, observations in the sheep experimental model indicate that BLV miRNAs contribute to leukemogenesis (Fig 5). Because sheep develop a leukemia/lymphoma that closely mimics natural pathogenesis, BLV miRNAs are speculated to be important mediators of oncogenesis in the bovine species. It should be mentioned that the reduced proviral load observed in the pBLV-ΔmiRNA-infected animals is likely to cause reduced pathogenicity. In fact, it is not possible to dissociate both parameters in vivo since development of leukemia positively correlates with proviral loads in the PBMCs [32,33]. Whether BLV miRNAs exert a direct oncogenic role by targeting specific cellular transcripts independently of viral replication remains to be demonstrated.

A role of viral miRNAs in pathogenesis has important consequences for the safety of an anti-BLV vaccine that is currently under development [34]. Considering the evidence reported here, a safe live-attenuated vaccine should also include a deletion of the miRNAs to limit the risk of disease. Another consequence of this report relates to human health, although this topic is still controversial [35,36]. Because viral (BLV miRNA B4) and cellular (miRNA-29) miRNAs share identical seed sequences, there is also a potential threat for zoonotic-induced oncogenesis, as recently suggested by the association of BLV and breast cancer [37].

## Materials and Methods

### Construction and inoculation of the miRNA-deleted provirus

Plasmid pBLV-WT (GenBank: JC613347.1) contains an infectious and pathogenic wild-type BLV provirus (strain 344). An isogenic molecular clone (pBLVΔ-miRNA) was constructed by deleting pBLV-WT of the miRNA coding region (nucleotides 6170 to 6736 according to the BLV reference genome NC_001414.1) (see S1 Text). Calves and sheep were inoculated by subcutaneous injection of pBLV-WT or pBLVΔ-miRNA as described in reference [38]. At regular intervals of time, heparinized blood was collected by jugular venipuncture and an aliquot of plasma was cryopreserved. PBMCs were isolated by Lymphoprep density gradient centrifugation (Stemcell technologies), resuspended in fetal calf serum containing 10% DMSO (Sigma-Aldrich) and frozen at -80°C or in liquid nitrogen.

### Transcriptomic analysis

The BLV sequences corresponding to the miRNA region (nucleotides 6170 to 6759 of the reference BLV genome NC_001414.1) were inserted into a lentiviral vector (pLenti6, Life technologies) to construct pLenti-miRNA. HEK 293T cells were transfected using lipofectamine 2000 (Life Technologies) with pLenti6 or pLenti-miRNA vectors together with pCAG-HIVgp and pCMV-VSV-G-RSV-Rev (provided by Masahiro Fujii, Niigata University, Japan). Forty-eight hours after transfection, the supernatant was collected and incubated with BL3 cells in presence of polybrene (10μg/ml). BL3 is a bovine B cell line with lymphoblastic morphology derived from a naturally occurred lymphosarcoma [39]. After selection with blasticidin (10μg/ml), correct miRNA expression in BL3-miRNA cells was verified by RT-qPCR. Total RNA was isolated from 3 independent batches of BL3 and BL3-miRNA cells using the miRNANeasy kit (Qiagen). In parallel, RNA was also extracted in triplicate from bovine PBMCs isolated from pBLV-WT, pBLV-ΔmiRNA and mock- infected calves (3 animals in each category). Proviral loads in 100 PBMCs of these animals were comparable: 4, 0.4 and 5.5 copies (pBLV-WT) and 2.3, 1.5 and 1.7 copies (pBLV-ΔmiRNA). Truseq Stranded mRNA libraries were prepared according to the manufacturer’s instructions (Illumina) and indexed 100bp paired-end runs were acquired on a HiSeq2000 Illumina sequencer. After mapping of the reads with the Tophat software, differential expression analysis was performed with Cufflinks.

### Construction of effector/sensor plasmids and luciferase reporter assays

Details on plasmid constructions are provided in the Supporting Information file S1 Text. After extraction of BL3 RNA, reverse transcription and PCR amplification, a series of luciferase reporter plasmids (ANXA1, GZMA, FOS, MAP2K1, PIK3CG and PPT1) were constructed by inserting the full-length bovine cDNA sequences into psiCHECK2 (Promega). Mutations of the predicted target sequences in these reporter plasmids were introduced using the Q5 directed mutagenesis kit (NEB). Reporter constructs (500ng) were transfected into 200,000 BL3 or BL3-miRNA cells using the Neon Transfection System (ThermoFisher Scientific). Electroporation parameters included 3 pulses of 1350V during 10ms. Effector plasmid pSUPER-miRNA was constructed by cloning the five miRNA hairpins (nucleotides 6170 to 6759 of the BLV reference genome NC_001414.1) into BglII and HindIII sites of expression vector pSUPER (Oligoengine). pSUPER-B4 only contains the miRNA-B4 hairpin (nucleotides 6484 to 6664). Reporter (100ng) and effector (500ng) constructs were transfected with Lipofectamine 2000 into 200,000 HEK 293T cells (293T/17; ATCC CRL-11268) in a 24 well plate. After 24 hours, cells were lysed and luciferase activities were measured using the Dual-Glo Luciferase Assay System (Promega) according to the manufacturer’s instructions. Assays were carried out in independent biological triplicates. See S1 Text for primers used for cloning.

### Quantification of proviral loads

DNA was extracted from PBMCs using DNeasy Blood and Tissue kit (Qiagen). BLV sequences were PCR amplified using pol gene sequence-specific primers 5'-GAAACTCCAGAGCAATGGCATAA-3' and 5'-GGTTCGGCCATCGAGACA-3'. As reference for quantification, β-actin was amplified with oligonucleotides 5'-TCCCTGGAGAAGAGCTACGA-3’ and 5’-GGCAGACTTAGCCTCCAGTG-3'. DNA was amplified by real-time quantitative PCR in a Roche light cycler using MESA green master mix (Eurogentec). The thermal protocol was initiated by a 5 min denaturation step at 95°C, followed by 45 cycles (15 sec at 95°C, 20 sec at 60°C, 40 sec at 72°C) and terminated by a melting curve. PCR efficiencies were calculated for each sample using 100ng, 33ng and 11ng of DNA. Standard curves were generated using PCR4topo vectors (Life Technologies) containing the corresponding pol or actin amplicon. Proviral load was calculated, as an average of the three dilutions, from the number of proviral copies divided by half of the number of actin copies and expressed as number of proviral copies per 100 of PBMCs.

### Nested PCR of proviral sequences

One hundred nanograms of genomic DNA were used for the first round of PCR using primers (Fw 5’-GCTTGACCTCTCGCCTTTTA-3’; Rv GTGCCGAGGTGGAAATAGAA), Phusion hot start II High Fidelity DNA polymerase and High Fidelity buffer (NEB). Thermal cycling conditions were: 30 sec at 98°C; 35 cycles (98°C 5 sec, 63°C 10 sec, 72°C 30 sec); 2 min at 72°C. One microliter of a 10-fold dilution of first round PCR was used as template for the second round PCR using primers (nFw 5’-CCCCTAAACCCGATTCTGAT-3’; nRv 5’-GGGCTTGTTACATGGGAAGA-3’). Thermal protocol for nested PCR were: 30 sec at 98°C, 35 cycles (5 sec at 98°C, 10 sec at 62°C, 30 sec at 72°C), 2 min at 72°C. Actin gene DNA was amplified with primers (Fw 5’-TCCCTGGAGAAGAGCTACGA-3’ and Rv 5’-GGCAGACTTAGCCTCCAGTG-3’) from 100 ng of genomic DNA according to the cycling protocol: 30 sec at 98°C followed by 35 cycles of (5 sec at 98°C /10 sec at 64°C / 30 sec at 72°C) and terminated by 2 min at 72°C.

### Quantification of viral mRNAs by RT-qPCR

PBMCs were cultivated during 24 hours in RPMI medium supplemented with L-glutamine, antibiotics, 10% fetal calf serum (FCS), 0.2μM of phorbol 12-myristate 13-acetate (PMA), 0.5μM of ionomycin, 0.1μM of concanamycin A (Sigma Aldrich). HEK293T were transfected with plasmids pBLV-WT or pBLVΔ-miRNA using lipofectamine 2000 (Life Technologies) and cultivated during 48 hours in RPMI medium supplemented with L-glutamine, antibiotics and 10% FCS. Total RNA was extracted from ex vivo cultivated PBMCs and transfected HEK293T using the miRNeasy kit (Qiagen). After digestion with Turbo DNAse (Life Technologies), cDNA was synthesized with random hexamers using the Reverse Transcriptase Superscript III (Life Technologies). cDNA was amplified by PCR with primers for HPRT (5’-GGTCAAGAAGCATAAACCAAAG-3’ and 5’-AAGGGCATATCCCACAACAAAC-3’), Tax/Rex (5’-GCGTTTGCTGAAAGCCTTCAA-3’ and 5’-GGGCAGGCATGTAGAGAGTG-3’), Gag (5’-TCCCTTTCTCATCACGTTCC-3’ and 5’-GTGGGGGTGAATGGTGTAAC-3’) and Env (5’-CTATCCGGCAGCGGTCAG-3’ and 5’-GAGGAGAGTAAGAGTGAGACTTACCC-3’) in a Roche light cycler using MESA green master mix (Eurogentec) according to the protocol: 5min denaturation at 95°C followed by 45 cycles (15 sec 95°C, 20 sec 60°C, 40 sec 72°C) and terminated with a melting curve. PCR efficiency was calculated for each sample using three serial dilutions of input DNA (100ng, 33ng and 11ng). Relative BLV/HPRT expression was calculated using the delta-delta Ct method.

### Quantification of cellular mRNAs by RT-qPCR

Total RNA was extracted from uncultured bovine PBMCs using the miRNeasy kit (Qiagen) and reversed transcribed into cDNA with the Reverse Transcriptase Superscript III (Life Technologies). Three cDNA dilutions (1x, 3x and 9x) were amplified by real-time quantitative PCR in a Roche light cycler using MESA green master mix (Eurogentec) according to the protocol: 5 min denaturation at 95°C followed by 45 cycles of PCR (15 sec at 95°C, 20 sec at 60°C, 40 sec at 72°C) and terminated with a melting curve. Primers used for PCR amplification are provided in the Supporting Information file S1 Text. After calculation of PCR efficiencies, mRNA expression relative to HPRT was using the delta-delta Ct method. cDNA was amplified for HPRT house-keeping gene transcript and for ANXA1, FOS, GZMA, MAP2K1, PIK3CG and PPT1.

### Quantification of BLV microRNA

Total RNA of HEK293T, YR2 and PBMCs was extracted using the miRNeasy kit (Qiagen), incubated with Turbo DNAse kit (Life Technologies) and reverse transcribed using the Taqman microRNA Reverse transcription kit (Life Technologies) and stem-loop custom BLV microRNA specific primers (Life Technologies). To generate HPRT cDNA, 1.5 pmol of primer 5’-AAGGGCATATCCCACAACAAAC-3’ was added at the end of the reverse transcription step and incubated for 10 min at 50°C. cDNA was PCR amplified in a Roche light cycler with HPRT primers (5’-GGTCAAGAAGCATAAACCAAAG-3’ and 5’-AAGGGCATATCCCACAACAAAC-3’) and custom Taqman BLV microRNA probes (Life Technologies) using MESA green master mix (Eurogentec) or Taqman Universal Master Mix II (Life Technologies), respectively. The thermal protocol used for microRNA quantification started with a denaturation step at 95°C for 10min followed by 45 PCR cycles (15 sec at 95°C, 60 sec at 60°C). For HPRT, PCR cycles included 5 min denaturation at 95°C followed by 45 cycles of PCR (15 sec at 95°C, 20 sec at 60°C, 40 sec at 72°C) and terminated with a melting curve. PCR efficiencies were calculated for each sample using three cDNA dilutions. Expression of BLV microRNA to relative to HPRT was calculated using the delta-delta Ct method.

### BLV microRNAs levels in plasma

Frozen plasmas were incubated for 10 min at 37°C, centrifuged during 1 hour at 20,000g and filtrated on 0.1μm pores. A spike-in control (C. elegans miR-39) was added to the plasma for absolute quantification according to the miRNeasy kit’s user manual. RNA was extracted using the miRNeasy Serum/Plasma kit (Qiagen) and reverse transcribed using the Taqman microRNA Reverse transcription kit (Life Technologies) with stem-loop custom microRNA primers (bovine let-7a and miR-92a, BLV miRNAs and C. elegans miR-39, Life Technologies). cDNA was synthesized using custom Taqman probe for each miR (Life Technologies). Three cDNA dilutions (1x, 3x and 9x) were amplified in a Roche light cycler using Taqman Universal Master Mix II (Life Technologies) according to the protocol: 10 min at 95°C followed by 45 PCR cycles (15 sec at 95°C, 60 sec at 60°C). Expression of BLV miRNAs relative to the miR-39 spike-in control was calculated by the using delta-delta Ct method.

### Ethics statement

Animal experimentation was conducted in accordance with the most recent national and international guidelines for animal care and use and following the directive 2010/63/UE of the European parliament. Handling of animals and experimental procedures were reviewed and approved by INTA´s Institutional Committee for Care and Use of Experimental Animals (CICUAE-INTA) under protocol number 35/2010 and by PIWet Committee for Care and Use of Experimental Animals under the protocol number 1515.

### Statistics

Statistical tests were performed using GraphPad Prism and Microsoft Excel. The symbols ***, **, * and NS were used when p<0.001, p<0.01, p<0.05 and Not Significant, respectively.

## Supporting Information

S1 Text The supporting file S1 Text contains supporting material and methods. (DOCX)

S1 Fig **Predicted interactions of BLV-miR-B4-3p with bovine cFOS (**A**), GZMA **(B)** and PPT1 **(C)** transcripts as determined with STarMir software.**
(DOCX)

S2 Fig Comparison of BLV miRNAs levels in transfected HEK293T cells, in primary PBMCs, in an ovine tumor cell line and in transduced BL3 lymphocytes. The amounts of B1-3p, B2-5p, B3-3p, B4-3p and B5-5p were measured by RT-qPCR in HEK 293T cells transfected with increasing amounts of plasmid pBLV-WT (500ng, 1 or 2 μg / 200,000 cells), in primary PBMCs isolated from 3 BLV cows with different proviral loads (2, 11 and 43 copies / 100 PBMCs), in a lymphocytic B cell line established from ovine PBMCs of a leukemic sheep (YR2) and in transduced BL3-miRNA cells. Error bars represent standard deviations.
(DOCX)

S3 Fig Specificity control of the psiCHECK2 luciferase reporter. **(A)** HEK293T cells were co-transfected with an empty psiCHECK2 luciferase reporter plasmid together with pSUPER, pSUPER-miRNAs or pSUPER-B4 expression vectors. Luciferase activity was determined at 24 hours post-transfection. **(B)** HEK 293T cells were co-transfected with a psiCHECK2 luciferase reporter plasmid containing the Mir-B4-3p complementary sequence together with pSUPER, pSUPER-miRNAs or pSUPER-B4 vectors. Luciferase activity was determined at 24 hours post-transfection. Statistical significance as determined by Student t-test: NS (not significant), * p< 0.05, ** p< 0.01, *** p< 0.001. Error bars represent standard deviations.
(DOCX)

S4 Fig BLV miRNA-associated GZMA down-regulation is restricted to the B cell lineage. GZMA mRNA levels were decreased in PBMCs and IgM+ cells from BLV WT compared to BLV-ΔmiRNA **(A and B)**, but not in B cell-depleted PBMCs **(C)**.
(DOCX)

S5 Fig Control of viral strain specificity in BLV-inoculated animals. DNA was isolated from primary PBMCs of 6 calves **(A)** and 6 sheep **(B)** infected with pBLV-WT (1–6 and 13–18) or pBLV-ΔmiRNA (7–12 and 19–24), as indicated. DNA sequences surrounding the miRNA cluster (see Fig 1A) were amplified by nested PCR.
(DOCX)

S6 Fig HBP1 expression is not affected by the BLV miRNAs. The transcriptomes of BL3 and BL3-miRNA cells were determined by RNA-Seq. HBP1 transcriptional activity is illustrated using the Integrative Genomics Viewer (IGV) software from the Broad Institute.
(DOCX)

S7 Fig Transduction of BLV miRNAs into the BL3 cell line was not associated with alterations of cell proliferation or apoptosis. **(A)** Cell proliferation was estimated by the decrease of CFSE intensity. No significant difference was observed between BL3 and BL3-miRNA cells. **(B)** Apoptotic cells were identified by AnnexinV/7AAD positivity (upper right quadrant). No significant difference was observed between BL3 and BL3-miRNA cells. Illustration of a representative experiment.
(DOCX)

S8 Fig HEK 293T cells were transfected with a vector containing wild-type (WT) or ΔmiRNA provirus and cultivated during 48h (experiment done in triplicate). **(A)** The levels of the viral RNAs (GAG, ENV and TAX) were determined by RT-qPCR. **(B)** The percentages of cells positive for the viral capsid protein p24 were determined by FACS. **(C)** The levels of capsid (CA, p24), envelope (SU, gp51) and Tax (p34) proteins were analyzed by western blot. Experiment done in triplicate. Statistical significance as determined by Student t-test, NS for not significant. Error bars represent standard deviations.
(DOCX)

S9 Fig Lack of transmission of the pBLV-ΔmiRNA strain from 2 dams to their calves. DNA was extracted from PBMCs of two calves born from pBLV-ΔmiRNA-infected dams and amplified by nested PCR. The absence of a 231bp fragment encompassing the ΔmiRNA region (Fig 1A) indicates the absence of pBLV-ΔmiRNA transmission. As control, genomic DNA integrity was supported by PCR of the actin gene.
(DOCX)
