# Supplementary material for: Bovine Leukemia Virus Small Noncoding RNAs Are Functional Elements That Regulate Replication and Contribute to Oncogenesis In Vivo
Source: PLoS Pathog. 2016 Apr 28;12(4):e1005588. doi: 10.1371/journal.ppat.1005588 (PMC4849745; doi:10.1371/journal.ppat.1005588)
Supplement: S1 Text — (DOCX) [file ppat.1005588.s001.docx]

**S1 Text: Supplementary Materials and Methods**

**Nucleotide sequences of wild-type and miRNA deleted proviruses**

Wild-type BLV

6139-ATCAACCTTCGACCCTGCCCTTGACACCCCCGTGTTTCACGCACCCTCAGGCTGTGGTGGGGCACTGGCTTAGTGGAATAGTCAGTGTACCATCACAAGCCTCTTCTTGCTGCCAGCACCGAGTTCGAACACAGCCCTACCCTGAGCCTCTCTGAGTACATGACTGAGTGTAGCGCAGAGAGGTTGTCGCTTCTGCGTGTCACTCAGTCATTTTTTATAGCCGATTGGGGTTCGCGCCCTCCCATTGCCTGTGACACGGTTAAGACCTCTCTCACTTCTGCTTCACCATCCCCCTGCCAGCGTTGGTCTAGTGGAAAGAACTAACGCTGACGGGGGCGATTTCTTGCAGCTGTGCTAAGCGAGAGGCTCTGGTGCTGGGGATAAGATGCGGCCCCTAGCACCACAGTCTCTGCGCCTTTTGGGTTCGAATCTTCCCCATGCAGCTTCCGCTTTTTACGCCCTGTTGCACACCCTTTCTAGAGATACCTGAAAATCTCAGCTCGCACCCCAAGGAAGGTTGTGGCTCAGAGGTTAAAATAGCTCGGACCGCAACCTCCCTTTCTTTTTATTCCACCCTCGCAAGGCCCCGGGTTCTGGGCCCCCTAACGGAGGTTCAGAATTTCCTCTACTAGGGGATGCTCAGGTCCAAGTGTGCACAATATCTCTTCCAAAAGGTCCTGATGAACATCTTCCCATGTAACAAGCCCCAGCAGAGACATTCC

-6860

Junction sequence at the miRNA-deletion

6139-ATCAACCTTCGACCCTGCCCTTGACACCCCCTCTAGACCCCCTAACGGAGGTTCAGAATTTCCTCTACTAGGGGATGCTCAGGTCCAAGTGTGCACAATATCTCTTCCAAAAGGTCCTGATGAACATCTTCCCATGTAACAAGCCCCAGCAGAGACATTCC

-6860

**Construction of effector and reporter luciferase plasmids**

Effector plasmid pSUPER-miRNA was constructed by cloning the five miRNA hairpins (nucleotides 6170 to 6759 of the BLV reference genome NC_001414.1) into BglII and HindIII sites of expression vector pSUPER (Oligoengine). pSUPER-B4 only contains the miRNA-B4 hairpin (nucleotides 6484 to 6664).

AGATCT: BglII restriction site

AAGCTT: HindIII restriction site

BLV_B4_BglII_psuper

CGATCAGATCTGCAGCTGTGCTAAGCGAGA

BLV_B4_HindIII_psuper

CGATCAAGCTTGAGCCACAACCTTCCTTGG

BLV_B12345_BglII_psuper

CGATCAGATCTGTGTTTCACGCACCCTCAG

BLV_B12345_HindIII_psuper

CGATCAAGCTTAATTCTGAACCTCCGTTAGGG

After extraction of BL3 RNA, reverse transcription and PCR amplification, a series of luciferase reporter plasmids (ANXA1, GZMA, FOS, MAP2K1, PIK3CG and PPT1) were constructed by inserting the full-length bovine cDNA sequences into psiCHECK2 (Promega). Mutations of the predicted target sequences in these reporter plasmids were introduced using the Q5 directed mutagenesis kit (NEB). Primers were the following:

Cloning of the full-length cDNAs (via AsisI and NotI restriction)

GCGATCGC: AsisI restriction site

GCGGCCGC: NotI restriction site

ANXA1_FL_AsisI_fw

AGTCAGCGATCGCCCTTCAAAAATGGCAATGGT

ANXA1_FL_NotI_rv

AGTCAGCGGCCGCAGGGTTTTATAGTTTGGAATAACCA

FOS_FL_ AsisI_fw

AGTCAGCGATCGCGCAAGCTTAAGGAGCTGACA

FOS_FL_ NotI_rv

AGTCAGCGGCCGCCAAGTTCACTTTCCACATGTCA

GZMA_FL_ AsisI_fw

AGTCAGCGATCGCCTTTCTGGCAGCCACTCTCT

GZMA_FL_ NotI_rv

AGTCAGCGGCCGCAAACGACAGCACTGGGTGTA

MAP2K1_FL_ AsisI_fw2

AGTCAGCGATCGCGCAGCTCAGAGGGAGGAAG

MAP2K1_FL_ NotI_rv

AGTCAGCGGCCGCGATTAGGCTTGTTAAACATCTGGA

PIK3CG_FL_ AsisI_fw2

AGTCAGCGATCGCGTCGCCTCTGCTGCTTTATC

PIK3CG_FL_ NotI_rv2

AGTCAGCGGCCGCCCTCCTCGCTCTTCCCTACT

PPT1_FL_ AsisI_fw

AGTCAGCGATCGCGCGGTCACGTGATAGAGTCA

PPT1_FL_ NotI_rv

AGTCAGCGGCCGCCCTTTCATCCCCTTGTTTCA

Mutagenesis of the full-length cDNA sequences (using Q5 site-directed mutagenesis)

Q5mut_FOS_mut_fw

AATTAACCTGacaCTGGATATTTTCAAATTGTATC

Q5mut_FOS_mut_rv

CCAATAACGAACCCAATAG

Q5mut1_GZMA_CDS_fw

CCTACATGGTatTACTTGATGGGG

Q5mut1_GZMA_CDS_rv

GTCTTGAATGAGGAGTCAC

Q5mut2_GZMA_3’UTR_fw

TGTTAAGCGGctCTGCAATGAAC

Q5mut2_GZMA_3’UTR_rv

ATAACCAGGACGTGGAAG

Q5mutsheep_PPT1_B4_fw

GCAGATGGTGtgGAATGCCAATGTAATTAG

Q5mutsheep_PPT1_B4_rv

TGACAGGGTGAGGCAAAG

Construction of luciferase reporters with predicted or mutated target sequences required primers:

Cloning of the predicted targets (via XhoI and NotI restriction)

GCGGCCGC: NotI restriction site

CTCGAG: XhoI restriction site

GATATC: EcoRV restriction site

Predicted target for BLV-mir-B4-3p on FOS mRNA

BLV-mir-B4-3p_vs_FOS_fw

TCGAGGATATCttattggaattaACCTGGTGCTgGC

BLV-mir-B4-3p_vs_FOS_rv

GGCCGCcAGCACCAGGTtaattccaataaGATATCC

Mutated predicted target

BLV-mir-B4-3p_vs_FOS_mut_fw

TCGAGGATATCttattggaattaACCTGACACTgGC

BLV-mir-B4-3p_vs_FOS_mut_rv

GGCCGCcAGTGTCAGGTtaattccaataaGATATCC

Predicted target for BLV-mir-B4-3p on GZMA mRNA (CDS)

BLV-mir-B4-3p_vs_GZMA(CDS)_fw

TCGAGGATATCctcattcaAGACCCTACATGGTGCTaGC

BLV-mir-B4-3p_vs_GZMA(CDS)_rv

GGCCGCtAGCACCATGTAGGGTCTtgaatgagGATATCC

Mutated predicted target

BLV-mir-B4-3p_vs_GZMA(CDS)_ mut_fw

TCGAGGATATCctcattcaAGACCCTACATGGTATTaGC

BLV-mir-B4-3p_vs_GZMA(CDS)_ mut_rv

GGCCGCtAATACCATGTAGGGTCTtgaatgagGATATCC

Predicted target for BLV-mir-B4-3p on GZMA mRNA (3’UTR)

BLV-mir-B4-3p_vs_GZMA(3’UTR)_fw

TCGAGGATATCTAGGTTGCTTCCACGTCCTGGTTATTGTTAAGCGGTGCTGCGC

BLV-mir-B4-3p_vs_GZMA(3’UTR)_rv

GGCCGCGCAGCACCGCTTAACAATAACCAGGACGTGGAAGCAACCTAGATATCC

Mutated predicted target

BLV-mir-B4-3p_vs_GZMA(3’UTR)_mut_fw

TCGAGGATATCTAGGTTGCTTCCACGTCCTGGTTATTGTTAAGCGGCTCTGCGC

BLV-mir-B4-3p_vs_GZMA(3’UTR)_mut_rv

GGCCGCGCAGAGCCGCTTAACAATAACCAGGACGTGGAAGCAACCTAGATATCC

Predicted target for BLV-mir-B4-3p on PPT1 mRNA

BLV-mir-B4-3p_vs_PPT1_fw

TCGAGGATATCaccctgtCAGCAGATGGTGCTgGC

BLV-mir-B4-3p_vs_PPT1_rv

GGCCGCcAGCACCATCTGCTGacagggtGATATCC

Mutated predicted target

BLV-mir-B4-3p_vs_PPT1_sheep_fw

TCGAGGATATCATCCTATCAGCAGATGGTGTGGGC

BLV-mir-B4-3p_vs_PPT1_sheep_rv

GGCCGCCCACACCATCTGCTGATAGGATGATATCC

Perfect target for BLV-mir-B4-3p

BLV-mir-B4-3p_perfect_target_Fw

TCGAGGATATCAAAGGCGCAGAGACCGTGGTGCTAGC

BLV-mir-B4-3p_perfect_target_Rev

GGCCGCTAGCACCACGGTCTCTGCGCCTTTGATATCC

**Primers for quantification of cellular mRNAs by RT-qPCR**

ANXA1_RTQPCR_fw

GGGCCTTGGGACTGATGAAG

ANXA1_RTQPCR_rv

CTCCAGATGTGTCTGAGGCG

FOS_RTQPCR_fw2

CATGGGTTCTCCCGTCAATG

FOS_RTQPCR_rv2

TGTTGGGATGAAGTTGGCACT

GZMA_RTQPCR_Fw

ATGGTGCTACTTGATGGGGG

GZMA_RTQPCR_Rv

GAGTGGGCCCCAAGAATGAT

MAP2K1_RTQPR_Fw

AGAGTTGGGAGCGCCTTTC

MAP2K1_RTQPR_Rv

TTCCGCTGCTGTTCATCGAG

PIK3CG_RTQPCR_fw

CTGCGCCAAGACATGCTTAT

PIK3CG_RTQPCR_rv

GTTCGTGCTGGTGGTTTCCT

PPT1_RTQPCR_fw

CTGGTGATCTGGCATGGGATG

PPT1_RTQPCR_rv

GCTGTTCTCCACATCCTCTCTG

**Depletion of IgM+ B cells from PBMCs**

PBMCs from BLV WT or ∆miRNA inoculated sheep were depleted from B cells using anti-IgM monoclonal IgG1 mouse antibody (clone 1H4) in association with immunomagnetic beads coated with rat anti-mouse IgG1 and MACS columns according to the manufacturer instructions (Miltenyi Biotec, Bergisch-Gladbach, Germany). Purity (less than 5% of IgM+ in the depleted fraction, more than 95% of IgM+ in the positive fraction) was evaluated by FACS analysis. GZMA mRNA levels were determined as described previsously.

**Proliferation of BL3 and BL3-miRNA cells in vitro**

BL3 and BL3-miRNA were labelled with CFSE according to the manufacturer instructions (eBioscience). Briefly, cells were labelled in PBS containing 1μM CFSE at 37°C during 10 minutes and washed with ice-cold RPMI medium. Cells were washed with RPMI medium and cultivated in RPMI 10%FCS with antibiotics. CFSE labelling was monitored every day by FACS analysis (BD FACS Calibur).

**Apoptosis of BL3 and BL3-miRNA cells in vitro**

Apoptosis of BL3 and BL3-miRNA cells was estimated using the AnnexinV-PE apoptosis detection kit I from BD biosciences according to the manufacturer instructions. Cells were analyzed by FACS (BD FACS Calibur) and percentage of AnnexinV+ 7AAD+ cells was measured.

**Expression of BLV mRNAs and BLV proteins in vitro**

HEK 293T were transfected with plasmids pBLV-WT or pBLV-∆miRNA using lipofectamine 2000 (Life Technologies) and cultivated during 48 hours in RPMI medium supplemented with L-glutamine, antibiotics and 10% FCS. Total RNA was extracted from cells using miRNeasy kit (Qiagen). Co-purified genomic DNA was removed using Turbo DNAse kit (Life Technologies). cDNAs were produced using Reverse Transcriptase Superscript III (Life Technologies) and random hexamers following manufacturer protocol. The following primers were used: HPRT (5’-GGTCAAGAAGCATAAACCAAAG-3’ and 5’-AAGGGCATATCCCACAACAAAC-3’), Tax/Rex (5’-GCGTTTGCTGAAAGCCTTCAA-3’ and 5’-GGGCAGGCATGTAGAGAGTG-3’), Gag/genomic (5’-TCCCTTTCTCATCACGTTCC-3’ and 5’-GTGGGGGTGAATGGTGTAAC-3’) and Env (5’-CTATCCGGCAGCGGTCAG-3’ and 5’-GAGGAGAGTAAGAGTGAGACTTACCC-3’). Three dilutions of cDNA were amplified by real-time quantitative PCR in a Roche light cycler using MESA green master mix (Eurogentec). The thermal protocol used started by a 95°C 5 min denaturation step; then 45 cycles as follow (95°C 15 sec, 60°C 20 sec, 72°C 40 sec) and terminated with a melting curve. PCR efficacies were calculated for each sample using the three dilutions. Relative BLV transcript expressions were calculated from HRPT expression using delta-delta Ct method. To determine the proportion of cells expressing the p24 viral protein, cells were fixed in 4% paraformaldehyde (Sigma Aldrich) and permeabilized using PBS-0.5% Triton X-100 (Sigma Aldrich). Intracellular detection of p24 was performed by sequential incubation with 4'G9 monoclonal antibody and a rat anti-mouse IgG1 Alexa488 conjugate (Life Technologies) for 30 min at 4 °C. Ten thousand events per sample were collected by flow cytometry (Becton Dickinson FACScalibur) and analyzed with the Cellquest software. To estimate the overall quantities of viral proteins p24 (CA), gp51 (SU) and p34 (Tax), cells were lyzed in RIPA buffer supplemented with the Complete protease inhibitor (Roche) during 30 min on ice. Protein concentration was measured by the Pierce BCA protein assay kit. Proteins (25μg) were resolved by SDS-PAGE and analyzed by Western blotting using standard procedures. Actin, p24, gp51 and Tax were detected with polyclonal rabbit anti-actin (Sigma Aldrich), anti-p24 4’G9, anti-SU BLV_2_ (VMRD) and anti-Tax 5A5 monoclonal antibodies, respectively. After incubation with HRP-conjugated anti-IgG secondary antibodies (Dako), immunoblots were revealed with the Pierce ECL western blotting substrate and a GE ImageQuant LAS imager.
