# Supplementary material for: Bovine Leukemia Virus Small Noncoding RNAs Are Functional Elements That Regulate Replication and Contribute to Oncogenesis In Vivo
Source: PLoS Pathog. 2016 Apr 28;12(4):e1005588. doi: 10.1371/journal.ppat.1005588 (PMC4849745; doi:10.1371/journal.ppat.1005588)
Supplement: S1 Fig — Predicted interactions of BLV-miR-B4-3p with bovine cFOS (A), GZMA (B) and PPT1 (C) transcripts as determined with STarMir software. (DOCX) [file ppat.1005588.s002.docx]

**Supplementary figure**

**S1 Fig.**

**S1 Fig.** Predicted interactions of BLV-miR-B4-3p with bovine cFOS (**A**), GZMA **(B)** and PPT1 **(C)** transcripts as determined with STarMir software.
