# Supplementary material for: Bovine Leukemia Virus Small Noncoding RNAs Are Functional Elements That Regulate Replication and Contribute to Oncogenesis In Vivo
Source: PLoS Pathog. 2016 Apr 28;12(4):e1005588. doi: 10.1371/journal.ppat.1005588 (PMC4849745; doi:10.1371/journal.ppat.1005588)
Supplement: S2 Fig — The amounts of B1-3p, B2-5p, B3-3p, B4-3p and B5-5p were measured by RT-qPCR in HEK 293T cells transfected with increasing amounts of plasmid pBLV-WT (500ng, 1 or 2 μg / 200,000 cells), in primary PBMCs isolated from 3 BLV cows with different proviral loads (2, 11 and 43 copies / 100 PBMCs), in a lymphocytic B cell line established from ovine PBMCs of a leukemic sheep (YR2) and in transduced BL3-miRNA cells. Error bars represent standard deviations. (DOCX) [file ppat.1005588.s003.docx]

**Supplementary figures**

**S2 Fig.**

**S2 Fig.** Comparison of BLV miRNAs levels in transfected HEK293T cells, in primary PBMCs, in an ovine tumor cell line and in transduced BL3 lymphocytes. The amounts of B1-3p, B2-5p, B3-3p, B4-3p and B5-5p were measured by RT-qPCR in HEK 293T cells transfected with increasing amounts of plasmid pBLV-WT (500ng, 1 or 2 µg / 200,000 cells), in primary PBMCs isolated from 3 BLV cows with different proviral loads (2, 11 and 43 copies / 100 PBMCs), in a lymphocytic B cell line established from ovine PBMCs of a leukemic sheep (YR2) and in transduced BL3-miRNA cells. Error bars represent standard deviations.
