# Supplementary material for: Bovine Leukemia Virus Small Noncoding RNAs Are Functional Elements That Regulate Replication and Contribute to Oncogenesis In Vivo
Source: PLoS Pathog. 2016 Apr 28;12(4):e1005588. doi: 10.1371/journal.ppat.1005588 (PMC4849745; doi:10.1371/journal.ppat.1005588)
Supplement: S3 Fig — (A) HEK293T cells were co-transfected with an empty psiCHECK2 luciferase reporter plasmid together with pSUPER, pSUPER-miRNAs or pSUPER-B4 expression vectors. Luciferase activity was determined at 24 hours post-transfection. (B) HEK 293T cells were co-transfected with a psiCHECK2 luciferase reporter plasmid containing the Mir-B4-3p complementary sequence together with pSUPER, pSUPER-miRNAs or pSUPER-B4 vectors. Luciferase activity was determined at 24 hours post-transfection. Statistical significance as determined by Student t-test: NS (not significant), * p< 0.05, ** p< 0.01, *** p< 0.001. Error bars represent standard deviations. (DOCX) [file ppat.1005588.s004.docx]

**Supplementary figures**

**S3 Fig.**

**S3 Fig.** Specificity control of the psiCHECK2 luciferase reporter. **(A)** HEK293T cells were co-transfected with an empty psiCHECK2 luciferase reporter plasmid together with pSUPER, pSUPER-miRNAs or pSUPER-B4 expression vectors. Luciferase activity was determined at 24 hours post-transfection. **(B)** HEK 293T cells were co-transfected with a psiCHECK2 luciferase reporter plasmid containing the Mir-B4-3p complementary sequence together with pSUPER, pSUPER-miRNAs or pSUPER-B4 vectors. Luciferase activity was determined at 24 hours post-transfection. Statistical significance as determined by Student t-test: NS (not significant), * p< 0.05, ** p< 0.01, *** p< 0.001. Error bars represent standard deviations.
