# Supplementary material for: Bovine Leukemia Virus Small Noncoding RNAs Are Functional Elements That Regulate Replication and Contribute to Oncogenesis In Vivo
Source: PLoS Pathog. 2016 Apr 28;12(4):e1005588. doi: 10.1371/journal.ppat.1005588 (PMC4849745; doi:10.1371/journal.ppat.1005588)
Supplement: S4 Fig — GZMA mRNA levels were decreased in PBMCs and IgM+ cells from BLV WT compared to BLV-ΔmiRNA (A and B), but not in B cell-depleted PBMCs (C). (DOCX) [file ppat.1005588.s005.docx]

**Supplementary figures**

**S4 Fig.**

**S4 Fig.** BLV miRNA-associated GZMA down-regulation is restricted to the B cell lineage. GZMA mRNA levels were decreased in PBMCs and IgM+ cells from BLV WT compared to BLV-∆miRNA **(A and B)**, but not in B cell-depleted PBMCs **(C)**.
