# Supplementary material for: Bovine Leukemia Virus Small Noncoding RNAs Are Functional Elements That Regulate Replication and Contribute to Oncogenesis In Vivo
Source: PLoS Pathog. 2016 Apr 28;12(4):e1005588. doi: 10.1371/journal.ppat.1005588 (PMC4849745; doi:10.1371/journal.ppat.1005588)
Supplement: S5 Fig — DNA was isolated from primary PBMCs of 6 calves (A) and 6 sheep (B) infected with pBLV-WT (1–6 and 13–18) or pBLV-ΔmiRNA (7–12 and 19–24), as indicated. DNA sequences surrounding the miRNA cluster (see Fig 1A) were amplified by nested PCR. (DOCX) [file ppat.1005588.s006.docx]

**Supplementary figures**

**S5 Fig.**

**S5 Fig.** Control of viral strain specificity in BLV-inoculated animals. DNA was isolated from primary PBMCs of 6 calves **(A)** and 6 sheep **(B)** infected with pBLV-WT (1-6 and 13-18) or pBLV-ΔmiRNA (7-12 and 19-24), as indicated. DNA sequences surrounding the miRNA cluster (see Figure 1A) were amplified by nested PCR.
