# Supplementary material for: Bovine Leukemia Virus Small Noncoding RNAs Are Functional Elements That Regulate Replication and Contribute to Oncogenesis In Vivo
Source: PLoS Pathog. 2016 Apr 28;12(4):e1005588. doi: 10.1371/journal.ppat.1005588 (PMC4849745; doi:10.1371/journal.ppat.1005588)
Supplement: S6 Fig — The transcriptomes of BL3 and BL3-miRNA cells were determined by RNA-Seq. HBP1 transcriptional activity is illustrated using the Integrative Genomics Viewer (IGV) software from the Broad Institute. (DOCX) [file ppat.1005588.s007.docx]

**Supplementary figures**

**S6 Fig.**

**S6 Fig.** HBP1 expression is not affected by the BLV miRNAs. The transcriptomes of BL3 and BL3-miRNA cells were determined by RNA-Seq. HBP1 transcriptional activity is illustrated using the Integrative Genomics Viewer (IGV) software from the Broad Institute.
