# Supplementary material for: Bovine Leukemia Virus Small Noncoding RNAs Are Functional Elements That Regulate Replication and Contribute to Oncogenesis In Vivo
Source: PLoS Pathog. 2016 Apr 28;12(4):e1005588. doi: 10.1371/journal.ppat.1005588 (PMC4849745; doi:10.1371/journal.ppat.1005588)
Supplement: S7 Fig — (A) Cell proliferation was estimated by the decrease of CFSE intensity. No significant difference was observed between BL3 and BL3-miRNA cells. (B) Apoptotic cells were identified by AnnexinV/7AAD positivity (upper right quadrant). No significant difference was observed between BL3 and BL3-miRNA cells. Illustration of a representative experiment. (DOCX) [file ppat.1005588.s008.docx]

**Supplementary figures**

**S7 Fig.**

**S7 Fig.** Transduction of BLV miRNAs into the BL3 cell line was not associated with alterations of cell proliferation or apoptosis. **(A)** Cell proliferation was estimated by the decrease of CFSE intensity. No significant difference was observed between BL3 and BL3-miRNA cells. **(B)** Apoptotic cells were identified by AnnexinV/7AAD positivity (upper right quadrant). No significant difference was observed between BL3 and BL3-miRNA cells. Illustration of a representative experiment.
