# Supplementary material for: Bovine Leukemia Virus Small Noncoding RNAs Are Functional Elements That Regulate Replication and Contribute to Oncogenesis In Vivo
Source: PLoS Pathog. 2016 Apr 28;12(4):e1005588. doi: 10.1371/journal.ppat.1005588 (PMC4849745; doi:10.1371/journal.ppat.1005588)
Supplement: S8 Fig — (A) The levels of the viral RNAs (GAG, ENV and TAX) were determined by RT-qPCR. (B) The percentages of cells positive for the viral capsid protein p24 were determined by FACS. (C) The levels of capsid (CA, p24), envelope (SU, gp51) and Tax (p34) proteins were analyzed by western blot. Experiment done in triplicate. Statistical significance as determined by Student t-test, NS for not significant. Error bars represent standard deviations. (DOCX) [file ppat.1005588.s009.docx]

**Supplementary figures**

**S8 Fig.**

**S8 Fig.** HEK 293T cells were transfected with a vector containing wild-type (WT) or ΔmiRNA provirus and cultivated during 48h. **(A)** The levels of the viral RNAs GAG, ENV and TAX were determined by RT-qPCR. **(B)** The percentages of cells positive for the viral capsid protein p24 were determined by FACS. **(C)** The levels of capsid (CA, p24), envelope (SU, gp51) and Tax (p34) proteins were analyzed by western blot. Experiment done in triplicate. Statistical significance as determined by Student t-test, NS for not significant. Error bars represent standard deviations.
