# Supplementary material for: Bovine Leukemia Virus Small Noncoding RNAs Are Functional Elements That Regulate Replication and Contribute to Oncogenesis In Vivo
Source: PLoS Pathog. 2016 Apr 28;12(4):e1005588. doi: 10.1371/journal.ppat.1005588 (PMC4849745; doi:10.1371/journal.ppat.1005588)
Supplement: S9 Fig — DNA was extracted from PBMCs of two calves born from pBLV-ΔmiRNA-infected dams and amplified by nested PCR. The absence of a 231bp fragment encompassing the ΔmiRNA region (Fig 1A) indicates the absence of pBLV-ΔmiRNA transmission. As control, genomic DNA integrity was supported by PCR of the actin gene. (DOCX) [file ppat.1005588.s010.docx]

**Supplementary figures**

**S9 Fig.**

**S9 Fig.** Lack of transmission of the pBLV-ΔmiRNA strain from 2 dams to their calves. DNA was extracted from PBMCs of two calves born from pBLV-ΔmiRNA-infected dams and amplified by nested PCR. The absence of a 231bp fragment encompassing the ΔmiRNA region (Figure 1A) indicates the absence of pBLV-ΔmiRNA transmission. As control, genomic DNA integrity was supported by PCR of the actin gene.
